# Knowledge attitudes and practice of breastmilk expression and storage among working mothers with infants under six months of age in Kenya

**DOI:** 10.1186/s13006-022-00469-6

**Published:** 2022-05-02

**Authors:** Priscillah Wanini Edemba, Grace Irimu, Rachel Musoke

**Affiliations:** Pediatrics and Child Health, The University of Nairobi, Nairobi, Kenya

**Keywords:** Lactation room, Working women, Workplace

## Abstract

**Background:**

Expression and storage of breastmilk is a strategy that ensures continued breast milk consumption in the event of temporary separation of an infant from the mother. However, many studies show that working mothers are unable to exclusively breastfeed for six months successfully. Working mothers are forced to wean early because of minimal support at the workplace, lack of knowledge on breast milk expression and lack of storage facilities. The 2017 Kenya Health Act mandates employers to provide lactation rooms for facilitation of breast milk expression in support of the lactating mother. This study analyses the knowledge attitude and practice of breast milk expression among working women in Kenya.

**Methods:**

This was a cross sectional study done between December 2018 and February 2019. Study participants were 395 working women with infants aged six months and below, attending well baby clinics in two large public hospitals in Nairobi Kenya. A structured questionnaire with open and closed ended questions was used to establish the knowledge and practice while a Likert scale was used to explore attitudes of the mothers towards expression and storage of breast milk.

**Results:**

Overall satisfactory knowledge on breast milk expression and storage was attained by only 34% of working mothers. Eighty four percent positively agreed that expression and storage of breast milk would help them achieve six months of exclusive breastfeeding. Challenges experienced were breast pain and cumbersome nature of expressing milk. Only 41% (161) were expressing breast milk either regularly or occasionally. The most common reason (24.7%) for expressing milk was to enable someone else feed the baby when they were at work. Most mothers (77%) expressed at home as the workplace did not seem to provide adequate equipment to facilitate breastmilk expression and storage.

**Conclusions:**

There is a substantial knowledge gap on expression and storage of breast milk. Working mothers have a good attitude towards attainment of exclusive breast milk feeding through expression of breast milk. The workplace does not have adequate facilities to support expression and storage of breast milk.

## Background

The 2016 *Lancet* series on breastfeeding estimates that scaling up breastfeeding to near universal levels would prevent 823,000 deaths in children under five years annually [1].The World Health Organization (WHO) recommends exclusive breastfeeding for the first six months of life. One of the World Health Assembly (WHA) global nutrition targets is to increase the rate of exclusive breastfeeding up to at least 70% by 2030 [2].

In Kenya, the exclusive breastfeeding rate rose from 32% in 2008 to 61% in 2014 according to the Kenya Demographic Health Survey (KDHS). However, there is a steady decline of exclusive breastfeeding rates from 3–5 months (63% at three months and 42% at five months) [3].

A quantitative study done in 2014 among the urban poor mothers in Kenya showed that expressing breast milk was not a common practice and was considered culturally unacceptable. Those who had attempted expressing breast milk said it was painful and total amount of milk expressed was low. There was also little knowledge on safe storage of breast milk, and they did not store milk due to the lack of refrigerators. Despite the mothers having a good general knowledge on exclusive breastfeeding recommendations, it did not translate into good practice due to work related issues. Support of breast milk expression is key to continuity of its consumption when mothers return back to work [4].

A 2018 study done among breastfeeding women in rural coastal Kenya showed that mothers and care givers had little knowledge and skills to express and store breast milk. There was resistance to the concept of storing breast milk and expression of milk was deemed important only in relieving engorgement after infant loss [5].

In 2016, a study done among working Kenyan women in an industrial town investigating constraints to breast milk expression showed that only 19% of the 85 working women were expressing milk. Reasons for not expressing milk included lack of knowledge on how to express breast milk (20%), lack of time to express milk (10%) as hand expression was time consuming, poor perception of the community on the practice of expressing breast milk and lack of breast pumps and fridges by 13% of the women [6].

The Academy of Breastfeeding Medicine clinical protocol #8 on human milk storage recommends the following safety storage conditions summarized in Table 1 [7].

**Table 1:** Summary of optimal storage conditions

	**Temperature (**^**0**^**C)**	**Maximum recommended storage duration**
Room	16–20	4 h optimal
		6–8 h acceptable
Refrigerator	~ 4	4 days optimal
		5–8 days under very clean conditions
Freezer	< -4	6 months optimal
		12 months acceptable

The Ministry of Health-Kenya launched guidelines for securing a baby friendly environment at the work place in 2018 that include a lactation room, breast milk expression breaks and a supportive work place policy to support attainment of exclusive breastfeeding among female employees [8]. The proposed lactation rooms and breast milk expression breaks will only benefit the working mother if she is conversant with how to utilize them for the purposes of achieving exclusive breast milk feeding. This study aims to examine the knowledge, attitudes, and practices related to breast milk expression and storage among working women in Kenya.

## Methods

This was cross-sectional study and data was collected between December 2018 and February 2019. Study sites were the well-baby clinics of Kenyatta National Hospital (KNH) and Mbagathi District Hospital (MDH), both situated in Nairobi county-Kenya. Only working mothers with infants aged six months and below were recruited after giving consent. The exclusion criteria included mothers below 18 years, those allowed to carry their infants to work and those with contraindications to breastfeeding according to the WHO criteria.

Sample size was calculated using the Fishers Formula where the expected value (*p*) was estimated at 50%, with a precision (d) of 5% giving a sample size of 384 participants.

Stratified sampling procedure was used to give a total of 299 mothers in KNH and 85 mothers in MDH.

A structured questionnaire was used to collect data. Contextualized Yes/No questions and open-ended questions were used to assess knowledge and practice respectively. A five-point Likert Scale was used to assess attitude. A soft copy interface of the questionnaire was developed using Epi Info software. The collected responses were stored in an excel format for analysis in R Studio Version 3.5.1 Software.

### Analysis of knowledge

A scoring system was used to analyze responses to knowledge-based closed ended questions: with 1 = correct response, 0 = Incorrect response (consistent with Academy of Breastfeeding Medicine protocol #8 revised in 2017) [7]. Any mother who did not know the answer was also considered to have an incorrect response. The midpoint score of total scores for each participant was used to distinguish between overall satisfactory and poor knowledge of breast milk expression and storage. Any total score above the midpoint was considered satisfactory. The level of knowledge was then cross tabulated against the variable of interest such as age, education, type of work and parity.

The variables were further analyzed using a multivariate analysis test to determine the factors independently associated with satisfactory knowledge. Associations between satisfactory knowledge and each independent variable were examined by Adjusted odds ratio (AOR) and 95% confidence interval.

Prior to regression, the collected data were explored through univariate data analysis of the independent variables to describe and find patterns within it. Univariate analysis of the continuous variables was done through calculation of measures of central tendencies. Univariate analysis of categorical variables was done through the calculation of proportions.

### Analysis of attitude

Responses for attitude were based on a five-point Likert scale. The responses were later collapsed into 3 cells representing agree, neutral and disagree for ease of interpretation. The results were then tabulated.

### Analysis of practice

Bivariate data analysis was done through regression of the binary outcome (expressing breast milk/ not expressing breast milk) against each independent variable. All independent variables that showed a statistically significant relation to the response variable in the bivariate regression analysis were used to develop a multiple regression model. All the analyses were done at an alpha value (critical *p*—value) of 0.05.

## Results

### Sociodemographic characteristics

A total of 395 working mothers were interviewed. The median age of the mothers was 29 years interquartile range (IQR) of 25–34 years. The median age of the infants when mother resumed work was three months (IQR = 3–6). More than half (52%) of the mothers had received tertiary level of education. In terms of workplace, the majority (76%) were in the private sector. At the time of data collection, only 41% of mothers were expressing breast milk either regularly or occasionally (Table 2).

**Table 2:** Sociodemographic characteristics of the participants (*n* = 395)

**Indicator**	**Median IQR**	
Age of mother (years)	29 (IQR = 25–34)	
Age of child (months)	4 (IQR = 3–6)	
Age of child when mother resumed work (months)	3 (IQR = 2–4)	
**Indicator**	**categories**	***n* (%)**
Gestational age of child at birth^a^	< = 36 weeks	77 (19.49)
	> 36 weeks	313 (79.24)
Highest level of education attained^a^	no formal education	6 (1.52)
	primary	44 (11.14)
	secondary	136 (34.43)
	tertiary	204 (51.65)
	not documented	5 (1.27)
Parity^a^	multiparous	252 (63.8)
	primiparous	138 (34.94)
Place of work^a^	private sector	302 (76.46)
	public sector	86 (21.77)
	not documented	7 (1.77)
Nature of mothers' employment^a^	salaried	175 (44.3)
	self employed	215 (54.43)
Expressing milk	no	234 (59.24)
	yes	161 (40.76)

^a^Minimal missing data

### Knowledge of breastmilk expression and storage

Almost all women (97%) had the correct knowledge that expression of milk could be done using hand or breast pump, but > 90% thought that there was a difference in contamination levels and volume of milk expressed between the various methods of expressing milk. Only 62% were aware that the Kenyan Government had directed employers to set up lactation stations at their workplaces (Table 3).

**Table 3:** Knowledge on breastmilk expression (1 = correct response, 0 = incorrect response)

**Indicator**	**Response**	**Score**	**Frequency n (%)**
What can be used to express breast milk?	not known	0	8 (2.03)
	hand or pump	1	387 (97.97)
	yes		
Is there any difference in volume when expressing by hand compared to pump?		0	353 (89.37)
	no	1	42 (10.63)
Is there any difference in contamination of milk when expressing by hand or pump?	yes	0	366 (92.66)
	no	1	29 (7.34)
Is it correct to discard the first few drops of milk before expressing milk?	yes	0	160 (40.51)
	no	1	235 (59.49)
Expressed milk is nutritious for an infant?	no	0	67 (16.96)
	yes	1	328 (83.04)
Is handwashing important before expressing breastmilk?	no	0	5 (1.27)
	yes	1	390 (98.73)
Is cleaning the breast important before expressing milk?	yes	0	365 (92.41)
	no	1	30 (7.59)
Has the government of Kenya directed employers to have lactation rooms?	no	0	149 (37.72)
	yes	1	246 (62.28)
Can breast milk can be stored at room temperature? (16-20^o^ C)	no	0	103 (26.08)
	yes	1	292 (73.92)
Can breastmilk be stored in refrigerator? (~ 4^o^ C)	no	0	75 (18.99)
	yes	1	320 (81.01)
Can breast milk be stored in a freezer? (< -4 ^o^ C)	no	0	162 (48.49)
	yes	1	228 (58.46)
How long can breast milk be stored in room temperature? (16-20^o^ C)	> 9 h	0	123 (31.14)
	8 h	1	272 (68.86)
How long breastmilk can be stored in a refrigerator? (~ 4^o^ C)	8 h or 9 months	0	242 (61.27)
	Up to 72 h	1	153 (38.73)
How long can breastmilk be frozen? (< 4 °C)	1 month or 24 h	0	309 (78.23)
	9 months	1	86 (21.77)
Is expressed breast milk nutritious if stored at room temperature? (16-20^o^ C)	no	0	84 (21.27)
	yes	1	311 (78.73)
Is expressed breast milk nutritious if stored in a refrigerator? (~ 4 °C)	no	0	115 (29.12)
	yes	1	280 (70.89)
Is expressed breast milk nutritious if frozen for 6 months? (< 4 °C)	no	0	173 (43.8)
	yes	1	222 (56.2)
Does refrigerated milk have a different smell from fresh breast milk?	no	0	186 (47.09)
	yes	1	209 (52.91)
Can baby refuse to take stored milk because of smell?	yes	0	153 (38.47)
	no	1	242 (61.27)

The majority of mothers (> 70%) correctly stated that expressed breast milk can be stored at room temperature or refrigerated. However, the majority were unsure about safe storage duration of expressed breast milk in a refrigerator (61%) and freezer (78%) (Table 3).

The midpoint 11 of the total scores was used to distinguish between satisfactory and poor knowledge of breast milk expression and storage. Therefore, a score of 11 points and above was considered satisfactory.

Satisfactory knowledge on breast milk expression was attained by 43% (170) of mothers while 47% (186) had satisfactory knowledge on storage. When combined, only 34% (135) of mothers had satisfactory knowledge on breastmilk expression and storage.

Working in the public sector and attaining a tertiary level of education was significantly associated with satisfactory knowledge on expression and storage of breastmilk (Table 3).

### Attitude towards breast milk expression and storage

The majority of working women (94%) agreed that breastmilk expression could be done using hand technique. Pain during breast milk expression was experienced by 50% of mothers and 66% thought it was cumbersome.

A large number (87%) of the mothers agreed that proper storage of breast milk would help them succeed in exclusive breastfeeding however, 76% of them thought it was an expensive venture. The workplace did not have adequate facilities to support breast milk expression as shown in Table 4 by 75% of the mothers (Table 4).

**Table 4:** Attitudes towards breast milk expression and storage

Breast milk expression/ storage	Agree (%)	I don`t know (%)	Disagree (%)
Other feeds should be introduced to the baby after 6 months	386 (97.72)	0 (0)	4 (1.01)
Breast milk expression can allow mothers to achieve exclusive breastfeeding for 6 months	333 (84.3)	12 (3.04)	44 (11.14)
Breast milk expression is painful	198 (50.13)	33 (8.35)	159 (40.25)
Breast milk expression is cumbersome/ fussy	260 (65.82)	15 (3.8)	115 (29.11)
Breast milk expression can be done at the workplace	229 (57.97)	9 (2.28)	152 (38.48)
Your workplace has facilities that support breast milk expression	91 (23.04)	3 (0.76)	296 (74.94)
Breast milk expression can be done by hand	373 (94.43)	4 (1.01)	13 (3.29)
Proper storage of breast milk can help achieve six months of exclusive breastfeeding	344 (87.09)	13 (3.29)	33 (8.35)
Stored breast milk is safe for infants to drink	301 (76.2)	19 (4.81)	70 (17.72)
Storing breast milk is expensive	301 (76.2)	19 (4.81)	70 (17.72)
Stored breast milk has less nutritional value compared to milk that baby feeds directly from the breast	177 (44.81)	31 (7.85)	182 (46.08)
It is safe to keep expressed breast milk for up to 8 h at room temperature (16–20 °C)	247 (62.53)	58 (14.68)	85 (21.52)
It is safe to freeze (< 4 °C) expressed breast milk for up to 9 months	101 (25.57)	86 (21.77)	203 (51.39)
I would like to know how to express and store breast milk	367 (92.91)	0 (0)	23 (5.82)

### Practice of breast milk expression and storage

The majority (63%), of working mothers were taught how to express breast milk by healthcare professionals. Hand expression was preferred by 50% of the mothers. Room temperature storage was practiced by 49% of the mothers, 34% preferred storing milk in the refrigerator and 16% stored milk by freezing. Baby bottles were used as the preferred storage containers by 55% of mothers. Those with refrigerators, at home were 76% and 35% at work, and 77% preferred expressing breastmilk at home. (Table 5).

**Table 5:** Factors associated with knowledge of breast milk expression and storage (multivariate analysis)

	**Term**	**AOR**	**95%CI**	***p*-value**
Age	mother's age	1.09	1.03, 1.14	0.001
	< 36 weeks	ref		
Gestation period	> 36 weeks	0.89	0.49, 1.63	0.701
	primary	ref		
Level of education	secondary	1.81	0.78, 4.61	0.188
	tertiary	4.47	2.01, 11.07	0.001
	multiparous	ref		
Parity	primiparous	1.1	0.63, 1.91	0.742
	private sector	ref		
Place of work	public sector	2.26	1.33, 3.85	0.003

Chi- square statistical analysis showed a statistically significant relationship between expression of breast milk and ownership of a refrigerator: *p*—value < 0.001.

When multivariate analysis was done, mothers with tertiary education (AOR 3.9; CI 1.96, 8.41) were significantly associated with expression and storage of breast milk (Table 6 and Table 7)

**Table 6:** Practice of breastmilk expression and storage

Indicator	Responses	*n* = 161 (%)
Learnt how to express breastmilk from^a^	friend	14 (8.7)
	healthcare provider	101 (62.73)
	mass media	10 (6.21)
	others	7 (4.35)
	relative	28 (17.39)
Methods for breast milk expression	both hand & pump	20 (12.42)
	electric Pump	12 (7.45)
	hand	81 (50.31)
	manual pump	48 (29.81)
Storage of breast milk^a^	freeze (< 4 °C)	25 (15.53)
	refrigerator(~ 4 °C)	55 (34.16)
	room temperature	78 (48.45)
	(16–20 °C)	
Location of breast milk expression^a^	both	31 (19.25)
	home	124 (77.02)
	work	4 (2.48)
Availability of refrigerator at home	no	39 (24.22)
	yes	122 (75.78)
Availability of refrigerator at work	no	104 (64.6)
	yes	57 (35.4)
Preferred container for storing breastmilk^a^	baby bottle	88 (54.66)
	others	23 (14.29)
	polythene bag	3 (1.86)
	special breast milk bags	36 (22.36)

^a^Minimal missing data

**Table 7:** Factors associated with expression and storage of breast milk

	**Term**	**Univariable model**			**Multivariable model**		
		**OR**	**95%CI**	**p.value**	**AOR**	**95%CI**	**p.value**
Age	mother's age	1	0.96, 1.03	0.905	-	-	-
Education level	primary	ref			ref		
	secondary	1.64	0.79, 3.64	0.203	1.6	0.76, 3.55	0.229
	tertiary	3.91	1.96, 8.41	0	3.66	1.81, 7.95	0.001
Place of work	private sector	ref					
	public sector	1.68	1.04, 2.73	0.035	1.32	0.8, 2.18	0.283
Gestation period	< 36 weeks	ref			ref		
	> 36 weeks	0.76	0.46, 1.25	0.277	-	-	-
Exclusive breastfeed	no	ref					
	yes	0.77	0.48, 1.22	0.263	-	-	-

### Reasons for expressing breast milk

**Fig. 1:**
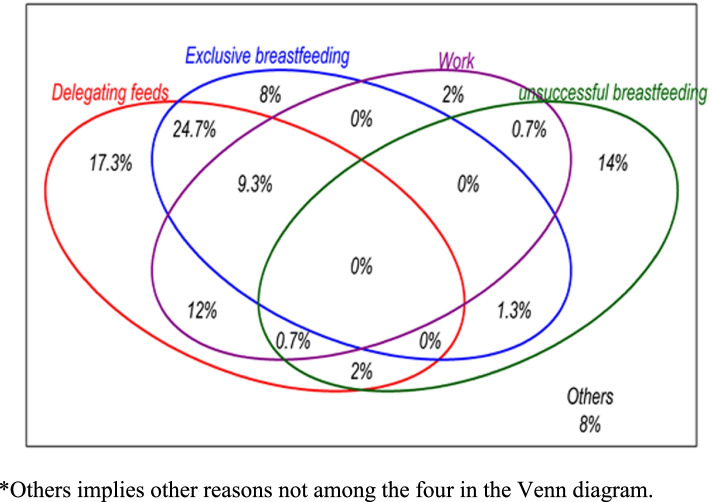
Reasons why mothers expressed and stored milk

The main reason why mothers expressed breastmilk was to enable them delegate feeding while they were at work (24.7%). Expression of breastmilk was also beneficial to those who had been unsuccessful at breastfeeding (14%) (Fig. 1).

## Discussion

The 2018 Baby Friendly Hospital Initiative guidelines recommend that at least 80% of mothers with preterm and term infants should correctly describe or demonstrate how to express milk [9]. The overall rate of satisfactory knowledge on both expression and storage of breastmilk was 34 percent. Satisfactory knowledge was more among those who expressed milk (47%) compared to those that did not at 25 percent. Acquiring tertiary education OR 4.5 (95% CI 2.01, 11.07) and working in the public sector OR 2.26 (95% CI 1.33, 3.85) was significantly associated with a possibility of having satisfactory knowledge. A 2016 study done in Saudi Arabia among 499 women showed that a higher education level was associated with more knowledge on breast milk expression (*p* value of < 0.001). This could have been due to their ability to access information on different fronts, access to better healthcare or financial strength to buy breast pumps and storage devices [10].

The attitudes towards expression and storage of breast milk were encouraging. Most participants (83%) knew that expressed breast milk retained its nutritional value and it would enable them achieve six months of exclusive breast milk feeding. In addition, 76% agreed that it was safe for infants to drink. This shows that working mothers are seeing an opportunity to achieve exclusive breastfeeding through expression and storage of breastmilk. However, 50% of our mothers felt that expression of breastmilk was painful and 66% thought it was cumbersome. A study done in Kenya among working women listed lack of time to express milk as one of the challenges associated with expression of breast milk. The technique of hand expression was perceived to be time consuming [6]. We did not explore knowledge of the manual technique of expression.

In our study, only 41% of the working mothers expressed and stored breastmilk which is higher than a 2016 Kenyan study at 19% [6] and a 2018 Nigerian study at 37 percent [11]. Of note is that each of these studies used different questions from ours and we focused on working, breastfeeding mothers with infants aged six months and below. Working mothers preferred to express their milk at home (77%) compared to expressing at work at 3%, yet 36% had access to a fridge at work. The majority of working mothers (75%) felt that there were inadequate facilities at work to support lactating mothers, yet the top reason (24.7%) for expressing milk was to achieve exclusive breastfeeding by delegating the task of feeding while at work. This concurs with a study done in Ghana among professional working women which showed that 69% of mothers received no additional support at work from their employers beyond maternity leave [12]. Besides providing lactation rooms, employers are encouraged to offer lactation support programs that facilitate lactation management during employment. They should also initiate mother friendly policies at the work place [13]. All employers have the ability to implement breastfeeding support programs that fit in the company budget and/ or resources [14].

Commendable effort has been made by the 2017 Kenyan Breastfeeding Mothers Act in defining lactation rooms. They should have a comfortable seat, a small table, electrical outlets, refrigeration facilities and they should not be located in the rest rooms [15].

## Conclusions

The study showed substantial knowledge gaps in expression and storage of breastmilk. Special emphasis needs to be put in place in all health facilities to educate mothers on expression and storage of breastmilk. Healthcare professionals with experience in lactation management should be available to assist any mother experiencing breast milk expression challenges. This will help them overcome painful episodes and the cumbersome attitude of expressing breast milk.

## Data Availability

The datasets used during the current study are available from the corresponding author on reasonable request.

## Abbreviations

ILO: International Labor Convention
IQR: Interquartile Range
KDHS: Kenya Demographic Health Survey
KNH: Kenyatta National Hospital
MDH: Mbagathi District Hospital
WHA: World Health Assembly
WHO: World Health Organization
UNICEF: United Nations International Children’s Emergency Fund
